# Is It Just Water Under the Bridge? An Eight-Day-Old With Late-Onset Group B Streptococcal Infection After Water Birth

**DOI:** 10.7759/cureus.8614

**Published:** 2020-06-14

**Authors:** Shannon A Solt, Rebecca L Smith, Afsaneh Pirzadeh, Tom Belhorn, Eric Zwemer

**Affiliations:** 1 Pediatric Critical Care Medicine, University of North Carolina at Chapel Hill, Chapel Hill, USA; 2 Pediatric Critical Care Medicine, UNC Hospitals, Chapel Hill, USA; 3 Pediatric Infectious Diseases, University of North Carolina at Chapel Hill, Chapel Hill, USA; 4 Pediatrics, University of North Carolina at Chapel Hill, Chapel Hill, USA

**Keywords:** water birth, sepsis, pediatrics, infectious diseases, gbs

## Abstract

Group B Streptococcus (Streptococcus agalactiae or GBS) infections are known as a leading cause of morbidity and mortality in the neonatal population. The role of water birth in colonizing and transmitting GBS between mother and infant is unclear. We present a case of an exclusively breastfed full-term infant, born via water birth, to a GBS-negative woman who developed GBS mastitis. The infant presented with severe, late-onset GBS meningitis/septic shock and subsequently developed fatal necrotizing enterocolitis. Literature regarding the potential role of water birth in GBS transmission is reviewed.

## Introduction

Group B Streptococcus (*Streptococcus agalactiae* or GBS) infections are known to be a leading cause of morbidity and mortality in the neonate [1]. Water birth has been associated with some improved maternal outcomes, but studies have shown the water, post water birth compared to laboring in water, has significantly higher colony density of GBS [2]. The incidence of neonatal GBS colonization following water birth, however, has not been found to be increased [2]. We describe a case of an exclusively breastfed full-term infant, born via water birth, to a GBS negative woman who developed GBS mastitis. We then review potential routes of GBS infection and transmission as they relate to water birth.

## Case presentation

An eight-day-old ex-full-term boy presented to the ER of an outside hospital for poor feeding. His mother had initially sought medical attention for her own mastitis, but she also informed providers that the child had not fed well that day and had decreased wet diapers. She denied the baby having any fevers, vomiting, rashes, diarrhea, and abnormal movements. Initial assessment revealed an ill- and gray-appearing infant with a temperature of 35.9°C, heart rate 201, respiratory rate 60, blood pressure 90/50, and oxygen saturation of 95% in room air. Given overall appearance and periods of apnea, the infant was intubated shortly after arrival. A full evaluation for sepsis was initiated, which was significant for a cerebrospinal fluid (CSF) white blood cell count of 139 cells/mm3 and a Gram stain showing Gram-positive cocci in clusters. He received 40 mL/kg of fluid resuscitation and was initiated on ampicillin and gentamicin before being transferred from the outside hospital ED to our pediatric ICU (PICU). 

The infant had been born full-term via waterbirth to a G1P1 mother with negative prenatal GBS testing. As such, no perinatal antibiotics had been given. Prenatal labs and ultrasounds were unremarkable, and there were no noted complications with the pregnancy, delivery, or postpartum stay. 

Upon admission to the PICU, the patient became hypotensive and was initiated on an epinephrine infusion. His blood and CSF cultures from the outside hospital returned positive for GBS within 12 hours. Given abnormal movements, a video electroencephalogram was placed and demonstrated frequent seizure activity. Seizures were treated with administration of phenobarbital, levetiracetam, and eventually continuous midazolam infusion. He continued to have refractory status epilepticus, and pentobarbital was initiated for burst suppression. Over the next few days, he stabilized and total parenteral nutrition, along with trophic feeds of maternal breast milk, was initiated. The patient’s mother continued to suffer from mastitis despite antibiotic therapy and therefore maternal breast milk cultures were sent. These cultures also returned positive for GBS, and enteral breastmilk feeds were discontinued. 

**Figure 1:**
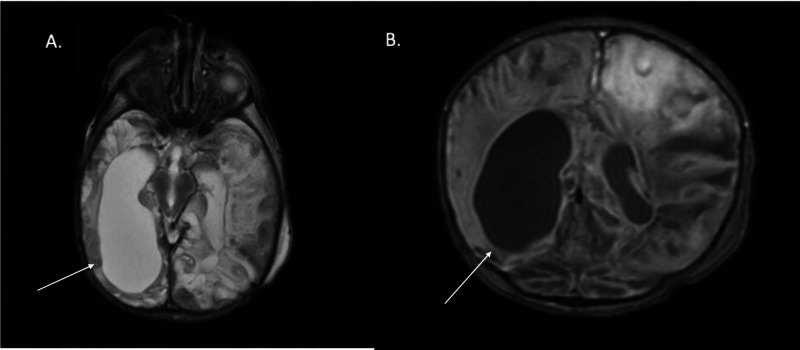
(A) T2 weighted axial image; (B) T1 coronal image. Both images show loss of significant brain tissue due to liquefactive necrosis of brain (arrows).

On hospital day (HD) five, the patient developed significant endocrinopathies, including diabetes insipidus, cerebral salt wasting, hypothyroidism, and adrenal insufficiency. Head ultrasound was concerning for a large fluid collection. He underwent brain MRI to assess the fluid collection, which showed striking loss of brain tissue and severe changes throughout, suggestive of liquefactive necrosis (Figure 1). 

 

**Figure 2:**
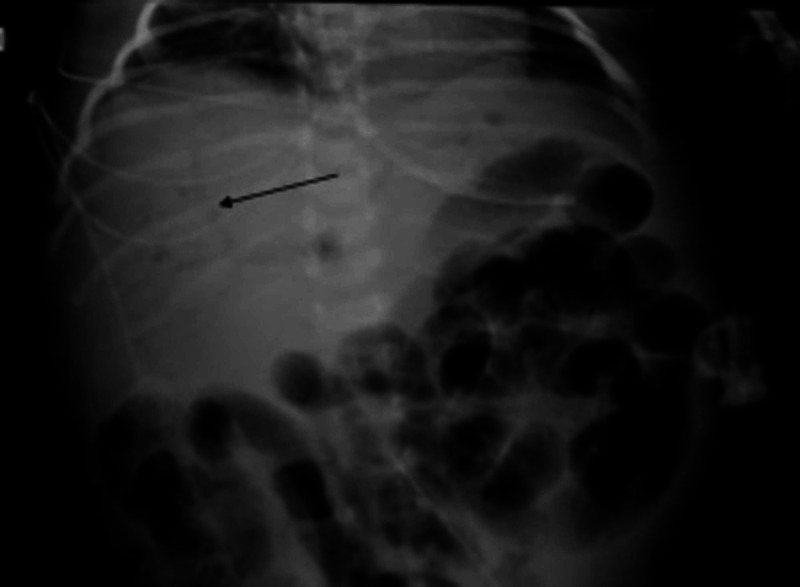
Abdominal X-ray showing portal venous gas (arrow).

On HD 16, enteral formula feeds were advanced to goal, and the patient clinically decompensated with large volume emesis and seizure activity. He became tachycardic and hypotensive, and subsequently developed a worsening metabolic acidosis. Feeds were stopped. Abdominal X-ray obtained showed portal venous gas but no pneumatosis (Figure 2). 

The patient was emergently taken to the operating room (OR). He was noted to have necrosis of bowel in an atypical distribution thought to be secondary to infarct. He had approximately 117 cm of bowel resected and the bowel was left in discontinuity. Hypercoagulable workup showed no significant findings. Two days later, the patient returned to the OR, and the remaining small bowel was noted to be necrotic and nonviable. After discussion with the family, life sustaining measures were withdrawn, and the infant passed away shortly thereafter. Autopsy and further hypercoagulable workup were unrevealing for any other contributing abnormalities. On autopsy, the bowel showed evidence of necrotizing enteritis but no evidence of direct bacterial invasion and was negative for mesenteric thromboemboli. 

## Discussion

Early-onset GBS infection manifests between birth and day six of life, while late-onset GBS infection occurs from day seven to three months of life. Early-onset infection rates have been dramatically reduced through the use of universal third trimester screening and initiation of widespread intrapartum antibiotic administration [1]. Late-onset GBS infection, however, is still a major problem, affecting 0.35 per 1000 live births [3]. While the pathogenesis of late-onset GBS infection is unknown, late-onset GBS disease has been associated with transmission via infected breast milk [4-5]. Infants with late-onset GBS infection commonly develop bacteremia and are more likely to develop meningitis [1]. Meningitis complicates approximately 15% of episodes of early-onset GBS sepsis and approximately 57% of late-onset GBS sepsis [6]. GBS is the most common cause of neonatal meningitis, and the mortality of GBS meningitis approaches 30% [6]. There are many reports in the literature of complications from GBS meningitis; however, most studies describe brain imaging consistent with abscesses, infarctions, arterial ischemic strokes, and/or cerebral sinovenous thrombosis [7]. Our patient developed liquefactive encephalomalacia as a consequence of his GBS meningitis. Only one other case report from Germany showed necrotizing or liquefactive encephalomalacia secondary to GBS meningitis [8]. Liquefactive necrosis occurs when the tissue in the brain, for unknown reasons, liquefies in response to ischemia [9]. 

Our case highlights the potential role of water birth in GBS infection. Water labor is considered by some practitioners to be a more natural way to give birth. It has some perceived benefits such as relaxation, diminished sensation of pain, decreased discomfort, and possibly a gentler birth experience for the newborn [10-11]. A prospective observational study of 1600 water births performed by Thoeni et al. showed water birth to be associated with a shorter first stage of labor, a lower episiotomy rate, and reduced analgesic requirements, with no increased risk of neonatal infections [12]. A prospective cohort study of 110 women, 32.7% of whom were GBS positive, showed that the bath water was significantly more colonized with GBS after water delivery (65% in the study group which consisted of women who labored and delivered in water compared to 25% in the control group which consisted of women who labored in water but delivered normally). Despite this, the incidence of neonatal GBS colonization was not significantly increased, even though 19% of the children in the study group and 31% of the children in the control group had positive nasal and/or pharyngeal GBS swabs [2]. One limitation of this study, however, is that the study group consisted of women who labored and delivered in water, while the control group included women who labored in water but delivered out of the water. This study did not compare GBS colonization of infants born to women with no water submersion at all. The conclusion from this study, and several others, is that water birth is not associated with a statistically significant increased risk of infections, as these studies have indicated that infants are not significantly more colonized with GBS post water birth [11, 13]. None of these studies, however, have examined whether mothers are more colonized with GBS after water birth. 

Testing for GBS is not infallible. Sensitivity is dependent on timing, collection and culture methods, and reported false negative rates that vary between nine percent and 50% [3]. In addition, some women may contract GBS after the initial third trimester screening. A study by Yancey et al. performed rectovaginal cultures on 826 women between 33 and 39 weeks’ gestation and then again at delivery and found that four percent of the women with negative antenatal cultures were colonized at delivery [14]. It is possible that the mother in this case was a GBS carrier who had a false negative test or contracted GBS later in her pregnancy. However, it is also possible that transmission through water birth may have occurred. It is possible that water contamination could occur from transmission from healthcare workers or family members assisting with the water birthing process [3].

## Conclusions

Late-onset GBS infection is associated with significant morbidity and mortality despite universal third trimester screening and intrapartum antibiotic prophylaxis. Water birth is an alternative method of delivery that has some perceived benefits for mother and infant. Current studies conclude that water birth is safe for newborns, but many of these studies have limitations, notably that most studies have focused on early-onset GBS infections and have failed to investigate maternal colonization with GBS after water birth. Our case illustrates an infant with late-onset GBS meningitis born to a prenatally GBS-negative mother who developed GBS mastitis. While it is impossible to know the exact GBS source for our patient, the course of events raise concern about whether water birth is as safe as previously described in the literature.
